# Supplementary material for: The effects of growth rate and biomechanical loading on bone laminarity within the emu skeleton
Source: PeerJ. 2019 Sep 25;7:e7616. doi: 10.7717/peerj.7616 (PMC6765378; doi:10.7717/peerj.7616)
Supplement: Table S1 — Vascular canals were counted in a sample area outlined by the periosteal extent of two bone fluorochromes in four octants representing the cardinal anatomical positions (wing elements: cranial, caudal, dorsal, ventral; hindlimb elements: cranial, caudal, lateral, medial). Laminarity Index (LI) measures the proportion of circular to total number of canals. [file peerj-07-7616-s001.docx]

| **Specimen** | **Element** | **Circular** | **Radial** | **Oblique** | **Longitudinal** | **Total** | **LI** |
| --- | --- | --- | --- | --- | --- | --- | --- |
| 15 | Femur | 4 | 2 | 0 | 113 | 119 | 0.03 |
|  | Tibiotarsus | 3 | 0 | 1 | 178 | 182 | 0.02 |
|  | Humerus | 6 | 0 | 1 | 22 | 29 | 0.21 |
|  | Ulna | 1 | 0 | 0 | 6 | 7 | 0.14 |
|  | Radius | 0 | 0 | 2 | 4 | 6 | 0 |
| 1c | Femur | 28 | 2 | 6 | 165 | 201 | 0.14 |
|  | Tibiotarsus | 19 | 1 | 11 | 127 | 158 | 0.12 |
|  | Humerus | 2 | 0 | 3 | 10 | 15 | 0.13 |
|  | Ulna | 0 | 0 | 1 | 2 | 3 | 0 |
|  | Radius | 1 | 0 | 0 | 8 | 9 | 0.11 |
| 17 | Femur | 6 | 2 | 3 | 374 | 385 | 0.02 |
|  | Tibiotarsus | 5 | 5 | 10 | 353 | 373 | 0.01 |
|  | Humerus | 11 | 2 | 3 | 22 | 38 | 0.29 |
|  | Ulna | 1 | 0 | 0 | 11 | 12 | 0.08 |
|  | Radius | 1 | 1 | 0 | 7 | 9 | 0.11 |
| 14b | Femur | 97 | 4 | 28 | 569 | 698 | 0.14 |
|  | Tibiotarsus | 64 | 24 | 34 | 336 | 458 | 0.14 |
|  | Humerus | 21 | 1 | 9 | 32 | 63 | 0.33 |
|  | Ulna | 4 | 1 | 3 | 13 | 21 | 0.19 |
|  | Radius | 3 | 2 | 7 | 8 | 20 | 0.15 |
| 16 | Femur | 202 | 2 | 21 | 199 | 424 | 0.48 |
|  | Tibiotarsus | 71 | 10 | 21 | 278 | 380 | 0.19 |
|  | Humerus | 30 | 0 | 5 | 17 | 52 | 0.58 |
|  | Ulna | 11 | 2 | 4 | 17 | 34 | 0.32 |
|  | Radius | 6 | 0 | 1 | 15 | 22 | 0.27 |
| 2a | Femur | 152 | 1 | 7 | 112 | 272 | 0.56 |
|  | Tibiotarsus | 106 | 8 | 15 | 174 | 303 | 0.35 |
|  | Humerus | 20 | 0 | 2 | 22 | 44 | 0.45 |
|  | Ulna | 1 | 1 | 0 | 2 | 4 | 0.25 |
|  | Radius | 0 | 0 | 0 | 3 | 3 | 0 |
| 21 | Femur | 26 | 1 | 7 | 56 | 90 | 0.29 |
|  | Tibiotarsus | 41 | 1 | 7 | 57 | 106 | 0.39 |
|  | Humerus | 28 | 8 | 12 | 81 | 129 | 0.22 |
|  | Ulna | 2 | 0 | 1 | 2 | 5 | 0.40 |
|  | Radius | 0 | 0 | 0 | 2 | 2 | 0 |
| 23 | Femur | 39 | 1 | 5 | 32 | 77 | 0.51 |
|  | Tibiotarsus | 31 | 1 | 4 | 17 | 53 | 0.58 |
|  | Humerus | 0 | 0 | 0 | 4 | 4 | 0 |
|  | Ulna | 0 | 1 | 0 | 1 | 2 | 0 |
|  | Radius | 0 | 0 | 0 | 1 | 1 | 0 |
