# Supplementary material for: The effects of growth rate and biomechanical loading on bone laminarity within the emu skeleton
Source: PeerJ. 2019 Sep 25;7:e7616. doi: 10.7717/peerj.7616 (PMC6765378; doi:10.7717/peerj.7616)
Supplement: Table S2 — Vascular canals were counted in a sample area outlined by the periosteal extent of two bone fluorochromes in the caudal octant only. Laminarity Index (LI) measures the proportion of circular to total number of canals. [file peerj-07-7616-s002.docx]

| **Specimen** | **Element** | **Circular** | **Radial** | **Oblique** | **Longitudinal** | **Total** | **Caudal Octant LI** |
| --- | --- | --- | --- | --- | --- | --- | --- |
| 15 | Femur | 0 | 2 | 0 | 27 | 29 | 0 |
|  | Tibiotarsus | 0 | 0 | 1 | 36 | 37 | 0 |
| 1c | Femur | 8 | 0 | 1 | 29 | 38 | 0.21 |
|  | Tibiotarsus | 5 | 1 | 2 | 30 | 38 | 0.13 |
| 17 | Femur | 0 | 1 | 0 | 56 | 57 | 0 |
|  | Tibiotarsus | 0 | 0 | 0 | 93 | 93 | 0 |
| 14b | Femur | 8 | 1 | 13 | 153 | 175 | 0.05 |
|  | Tibiotarsus | 11 | 6 | 12 | 155 | 184 | 0.06 |
| 16 | Femur | 34 | 2 | 8 | 85 | 129 | 0.26 |
|  | Tibiotarsus | 23 | 0 | 0 | 82 | 105 | 0.22 |
| 2a | Femur | 42 | 1 | 5 | 45 | 93 | 0.45 |
|  | Tibiotarsus | 36 | 0 | 3 | 22 | 61 | 0.59 |
| 21 | Femur | 6 | 1 | 6 | 28 | 41 | 0.15 |
|  | Tibiotarsus | 12 | 0 | 0 | 10 | 22 | 0.55 |
| 23 | Femur | 29 | 1 | 2 | 23 | 55 | 0.53 |
|  | Tibiotarsus | 17 | 1 | 1 | 2 | 21 | 0.81 |
