# Supplementary material for: The effects of growth rate and biomechanical loading on bone laminarity within the emu skeleton
Source: PeerJ. 2019 Sep 25;7:e7616. doi: 10.7717/peerj.7616 (PMC6765378; doi:10.7717/peerj.7616)
Supplement: Supplemental Information 1 [file peerj-07-7616-s004.docx]

Data deposition to accompany “The effects of growth rate and biomechanical loading on bone laminarity within the emu skeleton”

Paleohistology Repository (<http://paleohistology.appspot.com>)

| **Specimen** | **Element** | **Imaging mode** | **URL** |
| --- | --- | --- | --- |
| 15 | Humerus | Non-polarized light | <http://paleohistology.appspot.com/HTML5/15_H_100um.html> |
|  |  | Xylenol & Calcein | <http://paleohistology.appspot.com/HTML5/15_H_100um_XylCal.html> |
|  | Radius | Non-polarized light | <http://paleohistology.appspot.com/HTML5/15_R_100um.html> |
|  |  | Xylenol & Calcein | <http://paleohistology.appspot.com/HTML5/15_R_100um_XylCal.html> |
|  | Ulna | Non-polarized light | <http://paleohistology.appspot.com/HTML5/15_U_100um.html> |
|  |  | Xylenol & Calcein | <http://paleohistology.appspot.com/HTML5/15_U_100um_XylCal.html> |
|  | Femur | Non-polarized light | <http://paleohistology.appspot.com/HTML5/15_F_100um.html> |
|  |  | Xylenol & Calcein | <http://paleohistology.appspot.com/HTML5/15_F_100um_XylCal.html> |
|  | Tibiotarsus | Non-polarized light | <http://paleohistology.appspot.com/HTML5/15_TBT_100um.html> |
|  |  | Xylenol & Calcein | <http://paleohistology.appspot.com/HTML5/15_TBT_100um_XylCal.html> |
| 1c | Humerus | Non-polarized light | <http://paleohistology.appspot.com/HTML5/1c_H_100um.html> |
|  |  | Xylenol & Calcein | <http://paleohistology.appspot.com/HTML5/1c_H_100um_XylCal.html> |
|  | Radius | Non-polarized light | <http://paleohistology.appspot.com/HTML5/1c_R_100um.html> |
|  |  | Xylenol & Calcein | <http://paleohistology.appspot.com/HTML5/1c_R_100um_XylCal.html> |
|  | Ulna | Non-polarized light | <http://paleohistology.appspot.com/HTML5/1c_U_100um.html> |
|  |  | Xylenol & Calcein | <http://paleohistology.appspot.com/HTML5/1c_U_100um_XylCal.html> |
|  | Femur | Non-polarized light | <http://paleohistology.appspot.com/HTML5/1c_F_100um.html> |
|  |  | Xylenol & Calcein | <http://paleohistology.appspot.com/HTML5/1c_F_100um_XylCal.html> |
|  | Tibiotarsus | Non-polarized light | <http://paleohistology.appspot.com/HTML5/1c_TBT_100um.html> |
|  |  | Xylenol & Calcein | <http://paleohistology.appspot.com/HTML5/1c_TBT_100um_XylCal.html> |
| 17 | Humerus | Non-polarized light | <http://paleohistology.appspot.com/HTML5/17_H_100um.html> |
|  |  | Xylenol & Calcein | <http://paleohistology.appspot.com/HTML5/17_H_100um_XylCal.html> |
|  | Radius | Non-polarized light | <http://paleohistology.appspot.com/HTML5/17_R_100um.html> |
|  |  | Xylenol & Calcein | <http://paleohistology.appspot.com/HTML5/17_R_100um_XylCal.html> |
|  | Ulna | Non-polarized light | <http://paleohistology.appspot.com/HTML5/17_U_100um.html> |
|  |  | Xylenol & Calcein | <http://paleohistology.appspot.com/HTML5/17_U_100um_XylCal.html> |
|  | Femur | Non-polarized light | <http://paleohistology.appspot.com/HTML5/17_F_100um.html> |
|  |  | Xylenol & Calcein | <http://paleohistology.appspot.com/HTML5/17_F_100um_XylCal.html> |
|  | Tibiotarsus | Non-polarized light | <http://paleohistology.appspot.com/HTML5/17_TBT_100um.html> |
|  |  | Xylenol & Calcein | <http://paleohistology.appspot.com/HTML5/17_TBT_100um_XylCal.html> |
| 14b | Humerus | Non-polarized light | <http://paleohistology.appspot.com/HTML5/14b_H_100um.html> |
|  |  | Xylenol & Calcein | <http://paleohistology.appspot.com/HTML5/14b_H_100um_XylCal.html> |
|  | Radius | Non-polarized light | <http://paleohistology.appspot.com/HTML5/14b_R_100um.html> |
|  |  | Xylenol & Calcein | <http://paleohistology.appspot.com/HTML5/14b_R_100um_XylCal.html> |
|  | Ulna | Non-polarized light | <http://paleohistology.appspot.com/HTML5/14b_U_100um.html> |
|  |  | Xylenol & Calcein | <http://paleohistology.appspot.com/HTML5/14b_U_100um_XylCal.html> |
|  | Femur | Non-polarized light | <http://paleohistology.appspot.com/HTML5/14b_F_100um.html> |
|  |  | Xylenol & Calcein | <http://paleohistology.appspot.com/HTML5/14b_F_100um_XylCal.html> |
|  | Tibiotarsus | Non-polarized light | <http://paleohistology.appspot.com/HTML5/14b_TBT_100um.html> |
|  |  | Xylenol & Calcein | <http://paleohistology.appspot.com/HTML5/14b_TBT_100um_XylCal.html> |
| 16 | Humerus | Non-polarized light | <http://paleohistology.appspot.com/HTML5/16_H_100um.html> |
|  |  | Xylenol & Calcein | <http://paleohistology.appspot.com/HTML5/16_H_100um_XylCal.html> |
|  | Radius | Non-polarized light | <http://paleohistology.appspot.com/HTML5/16_R_100um.html> |
|  |  | Xylenol & Calcein | <http://paleohistology.appspot.com/HTML5/16_R_100um_XylCal.html> |
|  | Ulna | Non-polarized light | <http://paleohistology.appspot.com/HTML5/16_U_100um.html> |
|  |  | Xylenol & Calcein | <http://paleohistology.appspot.com/HTML5/16_U_100um_XylCal.html> |
|  | Femur | Non-polarized light | <http://paleohistology.appspot.com/HTML5/16_F_100um.html> |
|  |  | Xylenol & Calcein | <http://paleohistology.appspot.com/HTML5/16_F_100um_XylCal.html> |
|  | Tibiotarsus | Non-polarized light | <http://paleohistology.appspot.com/HTML5/16_TBT_100um.html> |
|  |  | Xylenol & Calcein | <http://paleohistology.appspot.com/HTML5/16_TBT_100um_XylCal.html> |
| 2a | Humerus | Non-polarized light | <http://paleohistology.appspot.com/HTML5/2a_H_100um.html> |
|  |  | Xylenol & Calcein | <http://paleohistology.appspot.com/HTML5/2a_H_100um_XylCal.html> |
|  | Radius | Non-polarized light | <http://paleohistology.appspot.com/HTML5/2a_R_100um.html> |
|  |  | Xylenol & Calcein | <http://paleohistology.appspot.com/HTML5/2a_R_100um_XylCal.html> |
|  | Ulna | Non-polarized light | <http://paleohistology.appspot.com/HTML5/2a_U_100um.html> |
|  |  | Xylenol & Calcein | <http://paleohistology.appspot.com/HTML5/2a_U_100um_XylCal.html> |
|  | Femur | Non-polarized light | <http://paleohistology.appspot.com/HTML5/2a_F_100um.html> |
|  |  | Xylenol & Calcein | <http://paleohistology.appspot.com/HTML5/2a_F_100um_XylCal.html> |
|  | Tibiotarsus | Non-polarized light | <http://paleohistology.appspot.com/HTML5/2a_TBT_100um.html> |
|  |  | Xylenol & Calcein | <http://paleohistology.appspot.com/HTML5/2a_TBT_100um_XylCal.html> |
| 21 | Humerus | Non-polarized light | <http://paleohistology.appspot.com/HTML5/21_H_100um.html> |
|  |  | Xylenol & Calcein | <http://paleohistology.appspot.com/HTML5/21_H_100um_XylCal.html> |
|  | Radius | Non-polarized light | <http://paleohistology.appspot.com/HTML5/21_R_100um.html> |
|  |  | Xylenol & Calcein | <http://paleohistology.appspot.com/HTML5/21_R_100um_XylCal.html> |
|  | Ulna | Non-polarized light | <http://paleohistology.appspot.com/HTML5/21_U_100um.html> |
|  |  | Xylenol & Calcein | <http://paleohistology.appspot.com/HTML5/21_U_100um_XylCal.html> |
|  | Femur | Non-polarized light | <http://paleohistology.appspot.com/HTML5/21_F_100um.html> |
|  |  | Xylenol & Calcein | <http://paleohistology.appspot.com/HTML5/21_F_100um_XylCal.html> |
|  | Tibiotarsus | Non-polarized light | <http://paleohistology.appspot.com/HTML5/21_TBT_100um.html> |
|  |  | Xylenol & Calcein | <http://paleohistology.appspot.com/HTML5/21_TBT_100um_XylCal.html> |
| 23 | Humerus | Non-polarized light | <http://paleohistology.appspot.com/HTML5/23_H_100um.html> |
|  |  | Xylenol & Calcein | <http://paleohistology.appspot.com/HTML5/23_H_100um_XylCal.html> |
|  | Radius | Non-polarized light | <http://paleohistology.appspot.com/HTML5/23_R_100um.html> |
|  |  | Xylenol & Calcein | <http://paleohistology.appspot.com/HTML5/23_R_100um_XylCal.html> |
|  | Ulna | Non-polarized light | <http://paleohistology.appspot.com/HTML5/23_U_100um.html> |
|  |  | Xylenol & Calcein | <http://paleohistology.appspot.com/HTML5/23_U_100um_XylCal.html> |
|  | Femur | Non-polarized light | <http://paleohistology.appspot.com/HTML5/23_F_100um.html> |
|  |  | Xylenol & Calcein | <http://paleohistology.appspot.com/HTML5/23_F_100um_XylCal.html> |
|  | Tibiotarsus | Non-polarized light | <http://paleohistology.appspot.com/HTML5/23_TBT_100um.html> |
|  |  | Xylenol & Calcein | <http://paleohistology.appspot.com/HTML5/23_TBT_100um_XylCal.html> |
